# Iron Biofortified Carioca Bean (*Phaseolus vulgaris* L.)—Based Brazilian Diet Delivers More Absorbable Iron and Affects the Gut Microbiota In Vivo (*Gallus gallus*)

**DOI:** 10.3390/nu10121970

**Published:** 2018-12-13

**Authors:** Desirrê Morais Dias, Nikolai Kolba, Dana Binyamin, Oren Ziv, Marilia Regini Nutti, Hércia Stampini Duarte Martino, Raymond P. Glahn, Omry Koren, Elad Tako

**Affiliations:** 1 Department of Nutrition and Health, Federal University of Viçosa, 36570000 Viçosa, Minas Gerais, Brazil; desirremorais@hotmail.com (D.M.D.); hercia72@gmail.com (H.S.D.M.); 2 Department of Food Science and Technology, Cornell University, Ithaca, NY 14850, USA; 3 USDA-ARS, Robert W. Holley Center for Agriculture and Health, Cornell University, Ithaca, NY 14850, USA; nikolai.kolba@ars.usda.gov (N.K.); raymond.glahn@ars.usda.gov (R.P.G.); 4 Azrieli Faculty of Medicine, Bar-Ilan University, Safed 1311502, Israel; dsimoni925@gmail.com (D.B.); oren.ziv@biu.ac.il (O.Z.); omry.koren@biu.ac.il (O.K.); 5 EMBRAPA Food Technology, 23020-470 Rio de Janeiro, Brazil; m.nuti@cgiar.org

**Keywords:** iron deficiency, Biofortification, intestinal morphometry, gut microbiome, metagenome, polyphenols

## Abstract

Biofortification aims to improve the micronutrient concentration and bioavailability in staple food crops. Unlike other strategies utilized to alleviate Fe deficiency, studies of the gut microbiota in the context of Fe biofortification are scarce. In this study, we performed a 6-week feeding trial in *Gallus gallus* (*n* = 15), aimed to investigate the Fe status and the alterations in the gut microbiome following the administration of Fe-biofortified carioca bean based diet (BC) versus a Fe-standard carioca bean based diet (SC). The tested diets were designed based on the Brazilian food consumption survey. Two primary outcomes were observed: (1) a significant increase in total body Hb-Fe values in the group receiving the Fe-biofortified carioca bean based diet; and (2) changes in the gut microbiome composition and function were observed, specifically, significant changes in phylogenetic diversity between treatment groups, as there was increased abundance of bacteria linked to phenolic catabolism, and increased abundance of beneficial SCFA-producing bacteria in the BC group. The BC group also presented a higher intestinal villi height compared to the SC group. Our results demonstrate that the Fe-biofortified carioca bean variety was able to moderately improve Fe status and to positively affect the intestinal functionality and bacterial populations.

## 1. Introduction

Micronutrients deficiency affects approximately two billion people worldwide. Iron (Fe) deficiency is the most prevalent nutrient deficiency, affecting around 40% of the world population, particularly women and children in developing countries [1,2]. It is estimated that around 46% of the population in Africa, 57% in South-East Asia and 19% in America are anemic [3]. Fe deficiency is highly prevalent in low-income countries (~30% in Brazil) due to a lack of meat consumption in addition to a notable dietary reliance on grains containing high amounts of Fe absorption inhibitors (e.g., phytic acid, polyphenolic compounds) [4,5,6,7]. Major pathophysiological complications related to insufficient Fe intake may include stunted growth, impaired physical and cognitive development, and increased risk of morbidity and mortality in children [4,8,9]. To alleviate Fe deficiency, an integral step involves the understanding of specific dietary patterns and components that contribute to Fe status in the particular population suffering from a deficiency.

Biofortified staple food crops have become an effective tool by which to address micronutrient deficiencies, especially that of Fe, in many at-risk populations [4,6,10,11]. The common bean (*Phaseolus vulgaris*) is one of the crops target for biofortification program since it exhibits sufficient genetic variability in iron concentration, which is the basic requirement for biofortification [12,13]. This crop is currently estimated to be one of the most important legumes worldwide [13,14], and is an important source of nutrients for more than 300 million people in parts of Eastern Africa and Latin America, representing 65% of total protein consumed, 32% of energy, and a major source of micronutrients (vitamins and minerals) [10,14,15].

Previous studies using Fe biofortified beans in Mexico [16], and Rwanda [17,18] have shown some improvement in Fe status in subjects consuming the biofortified beans versus a standard bean variety. However, a major challenge associated with biofortification of staple food crops, especially common beans, is that they contain factors such as polyphenols and phytic acid that can inhibit Fe bioavailability and absorption, hence limit their nutritional benefit [17,19]. These inhibitory factors may increase with Fe concentration when these crops are biofortified via conventional breeding [17,19,20]. Hence, as was previously suggested, it is necessary to measure the concentration of Fe, the amount of bioavailable Fe, and the concentration of potential inhibitors of Fe bioavailability in these biofortified crops [19,21,22]. It is also important to factor in and assess the other components of the diet in which these crops are consumed as the potential interactions can negate or even enhance the expected benefit of increased Fe content.

Despite containing inhibitory factors, legumes also carry other substances, referred to as promoters, which have the potential to counteract the effects of the inhibitory factors [19,20,23,24]. One of the most notable promoters are prebiotic [19,25,26]. Prebiotics have been characterized as a group of carbohydrates that resist digestion and absorption in the gastrointestinal tract (small intestine), that beneficially affect gut health, by enhancing the growth and activities of probiotics [26,27,28] and can improve mineral absorption [29]. These compounds can survive the acidic and enzymatic digestion in the small intestine, and thus can be fermented by probiotics that reside in the colon/cecum [30]. The fermentation of prebiotics by probiotics leads to the production of short-chain fatty acids (SCFA), which may improve the intestinal function, increasing the absorption of minerals such as Fe [25,31,32,33]. At the same time, some polyphenols present in the common beans can stimulate the growth of commensal and beneficial microbiota while pathogenic strains are inhibited or unaffected [34].

Biofortified crops have become an effective tool by which to address micronutrient deficiencies, especially that of Fe, in many at-risk populations [15,35,36]. By using the combination of a Caco-2 cell bioassay and an in vivo (*Gallus gallus*) model that has been used extensively for nutritional research and shown to be an excellent model to assess dietary Fe (and Zinc) bioavailability [4,37,38,39], the objective of the current study was to evaluate the ability of the Fe biofortified carioca bean line to deliver more Fe for hemoglobin (Hb) synthesis. Also, we aimed to evaluate the effect of the Fe biofortified carioca bean intake on the intestinal microbiota composition and function. If this in vivo assessment indicates that nutritional benefit exists, we suggest to further employ these screening tools to guide future studies aimed to assess biofortified staple food crops, as this approach will allow proceeding to human efficacy studies with greater confidence and success.

## 2. Materials and Methods 

### 2.1. Sample Preparation

The two carioca bean lines: BRS Perola (Fe Standard) and BRS Cometa (Fe Biofortified) that were used in this study were obtained from Embrapa (Empresa Brasileira de Pesquisa Agropecuaria, Goias, Brazil), and were shipped to Ithaca, New York in sealed containers imported as flours. The beans were cooked in three replicates in a conventional pressure cooker for 40 min using a bean/distilled water ratio of 1:2.7 (*w*/*v*) and dried in an air oven for 17 h at 60 °C. The dried beans were ground by stainless steel mill 090 CFT at 3000 rpm and stored at −12 °C [40].

### 2.2. Polyphenols Analysis

#### 2.2.1. Polyphenol Extraction

1 g of bean flour was added with 5 mL of methanol/water (50:50 *v*/*v*). The slurry was vortexed for 1 min, placed in a 24 °C sonication water bath for 20 min, vortexed again for 1 min and centrifuged at 4000× *g* for 15 min. The supernatant was filtered with a 0.45 μm Teflon syringe, filtered, and stored for later use at −20 °C. 

#### 2.2.2. Ultra Performance Liquid Chromatography—Mass Spectrometry (UPLC—MS) Analysis of Polyphenols

Extracts and standards were analyzed with a Waters Acquity UPLC (Waters, Milford, MA, USA). Five microliter samples were injected and passed through an Acquity UPLC BEH Shield RP18, 1.7 μm. 2.1 × 100 mm column (Waters, Milford, MA, USA) at 0.5 mL/min. The column was temperature-controlled at 40 °C. The mobile phase consisted of water with 0.1% formic acid (solvent A) and acetonitrile with 0.1% formic acid (solvent B). Polyphenols were eluted using linear gradients of 86.7–84.4% A in 1.5 min, 84.4–81.5% A in 0.2 min, 81.5–77% A in 2.8 min, 77–55% A in 0.5 min, 55–46% A in 1 min and 46–86.7% A in 0.2 min and a 0.8 min hold at 86.7% A for a total 7 min run time. From the column flow was directed into a Waters Acquity photodiode array detector set at 300–400 nm and a sampling rate of 20/s. Flow was then directed into the source of a Xevo G2 QTOF mass spectrometer (Waters, Milford, MA, USA) and ESI mass spectrometry was performed in negative ionization mode with a scan speed of 5/s in the mass range from 50 to 1200 Da. Capillary and cone gas voltages were set at 2.3 kV and 30 V respectively. Desolvation gas flow was 800 L/h. and desolvation gas temperature was 400 °C. Source temperature was 140 °C. Lock-mass correction was used with leucine encephalin as the lock-mass standard and a scan frequency of 25 s. Instrumentation and data acquisition were controlled by MassLynx software (version 4.2, Waters, Milford, MA, USA). Individual polyphenols in bean samples were tentatively determined by mass using MarkerLynx software (Waters, Milford, MA, USA), and their identities were confirmed by comparison of LC retention times with authentic standards. Polyphenol standard curves for flavonoids were derived from integrated areas under UV absorption peaks from 10 replications. Standard curves for catechin and 3.4-dihydroxybenzoic acid were constructed from MS ion intensities using 10 replications.

### 2.3. Phytate Analysis

Dietary phytic acid (phytate)/total phosphorus was measured as phosphorus released by phytase and alkaline phosphatase, following the kit manufacturer’s instructions (*n* = 5) (K-PHYT 12/12. Megazyme International. Bray, Ireland).

### 2.4. Iron Content of Bean Flour, Serum and Liver

The bean flour samples and liver samples (0.5 g) and serum (100 μL) were treated with 3.0 mL of 60:40 HNO_3_ and HClO_4_ mixture into a Pyrex glass tube and left for overnight to destroy organic matter. The mixture was then heated to 120 °C for two hours and 0.25 mL of 40 µg/g Yttrium (Sigma-Aldrich, St. Louis, MO, USA) added as an internal standard to compensate for any drift during the subsequent inductively coupled plasma atomic emission spectrometer (ICP-AES) analysis. The temperature of the heating block was then raised to 145 °C for 2 h. Then, the temperature of the heating block raised to 190 °C for ten minutes and turned off. The cooled samples in the tubes were then diluted to 20 mL, vortexed and transferred into auto sample tubes to analyze via ICP-AES. The model of the ICP used was a Thermo iCAP 6500 series (Thermo Jarrell Ash Corp., Franklin, MA, USA).

### 2.5. Protein and Dietary Fiber Analysis in the Bean Flour

Protein concentration was determined by micro-Kjeldahl method according to the Official Methods of Analysis (AOAC International, Rockville, MD, USA) procedure [41]. The determination of total fiber and soluble and insoluble fractions was performed by the enzymatic-gravimetric method according to AOAC [41], using the enzymatic hydrolysis for a heat-resistant amylase, protease and amyloglucosidase (Total dietary fiber assay Kiyonaga, Sigma^®^, Kawasaki, Japan).

### 2.6. In Vitro Iron Bioavailability Assessment

An established in vitro digestion/Caco-2 cell culture model was used to assess Fe-bioavailability [37,42]. The staple food flour samples (biofortified and standard beans, rice, potato) were analyzed by themselves and in a food combination (“food basket”). With this method, the cooked bean samples, additional meal plan components and the formulated diets were subjected to simulated gastric and intestinal digestion. 0.5 g of the freeze dried cooked beans and diet samples were utilized for each replication (*n* = 6) of the in vitro digestion [11,21,43].

### 2.7. Harvesting of Caco-2 Cells for Ferritin Analysis

The protocols used in the ferritin and the total protein contents analyses of Caco-2 cells were similar to those previously described [19,22,23,37,39,44]. Caco-2 cells synthesize ferritin in response to increases in intracellular Fe concentration. Therefore, we used the ratio of ferritin/total protein (expressed as ng ferritin/mg protein) as an indicator of cellular Fe uptake. All glassware used in the sample preparation and analyses was acid washed.

### 2.8. Animals, Diets and Study Design

Cornish cross—fertile broiler eggs (*n* = 60) were obtained from a commercial hatchery (Moyer’s chicks, Quakertown, PA, USA). The eggs were incubated under optimal conditions at the Cornell University Animal Science poultry farm incubator. Upon hatching (hatchability rate = 50%), chicks were allocated into 2 treatment groups on the basis of body weight and blood hemoglobin concentration (aimed to ensure equal concentration between groups), (1) Fe-standard carioca bean based diet (SC): 42% carioca bean (BRS Perola) based diet (*n* = 14), and (2) Fe-biofortified carioca bean based diet (BC): 42% carioca bean (BRS Cometa) based diet (*n* = 14). Experimental diets (Table 1) had no supplemental Fe. The specific Brazilian dietary formulation used in the study (Table 1) was based on the Brazilian food consumption survey [45]. Chicks were housed in a total confinement building (4 chicks per 1 m^2^ metal cage). The birds were under indoor controlled temperatures and were provided 16 h of light. Each cage was equipped with an automatic nipple drinker and a manual self-feeder. All birds were given ad libitum access to water. Feed intakes were measured daily (as from day 1), and Fe intakes were calculated from feed intakes and Fe concentration in the diets. The body weight and the hemoglobin concentration in the blood were measured weekly.

### 2.9. Blood Analysis, Hemoglobin (Hb) Determination, and Tissue Collection

Blood samples were collected weekly from the wing vein (100 μL) using micro-hematocrit heparinized capillary tubes (Fisher, Pittsburgh, PA, USA). Weekly blood Hb concentrations were determined spectrophotometrically using the Triton/NaOH method following the kit manufacturer’s instructions. Fe bioavailability was calculated as hemoglobin maintenance efficiency (HME):

$$\mathrm{HME}=\frac{\mathrm{Hb}-\mathrm{Fe},\;\mathrm{mg}\;\left(\mathrm{final}\right)–\mathrm{Hb}-\mathrm{Fe},\;\mathrm{mg}\;\left(\mathrm{initial}\right)}{\mathrm{Total}\;\mathrm{Fe}\;\mathrm{intake},\;\mathrm{mg}}\times 100$$

where Hb-Fe (index of Fe absorption) = total body hemoglobin Fe. Hb-Fe was calculated from hemoglobin concentrations and estimates of blood volume based on body weight (a blood volume of 85 mL per kg body weight is assumed):

Hb-Fe (mg) = BW (kg) × 0.085 blood/Kg × Hb (g/L) × 3.35 mg Fe/g Hb

At the end of the experiment (day 42), birds were euthanized by CO_2_ exposure. The digestive tracts (small intestine and cecum) and livers were quickly removed from the carcass. The samples were immediately frozen in liquid nitrogen, and then stored in a −80 °C freezer until further analysis.

All animal protocols were approved by the Cornell University Institutional Animal Care and Use Committee (protocol name: Intestinal uptake of Fe and Zn in the duodenum of broiler chicken: extent, frequency, and nutritional implications; approved: 15 December 2016; protocol number: 2007–0129).

### 2.10. Isolation of Total RNA from Chicken Duodenum and Liver

Total RNA was extracted from 30 mg of the proximal duodenal tissue (*n* = 8) and liver (*n* = 8) using Qiagen RNeasy Mini Kit (RNeasy Mini Kit, Qiagen Inc., Valencia, CA, USA) according to the manufacturer’s protocol. Briefly, tissues were disrupted and homogenized with a rotor-stator homogenizer in buffer RLT^®^, containing β-mercaptoethanol. The tissue lysate was centrifuged for 3 min at 8000× *g* in a micro centrifuge. An aliquot of the supernatant was transferred to another tube, combined with 1 volume of 70% ethanol and mixed immediately. Each sample (700 μL) was applied to an RNeasy mini column, centrifuged for 15 s at 8000× *g*, and the flow through material was discarded. Next, the RN easy columns were transferred to new 2-mL collection tubes, and 500 μL of buffer RPE^®^ was pipetted onto the RNeasy column followed by centrifugation for 15 s at 8000× *g*. An additional 500 μL of buffer RPE were pipetted onto the RNeasy column and centrifuged for 2 min at 8000× *g*. Total RNA was eluted in 50 μL of RNase free water.

All steps were carried out under RNase free conditions. RNA was quantified by absorbance at A 260/280. Integrity of the 28S and 18S ribosomal RNAs was verified by 1.5% agarose gel electrophoresis followed by ethidium bromide staining. DNA contamination was removed using TURBO DNase treatment and removal kit from AMBION (Austin, TX, USA).

### 2.11. Real Time Polymerase Chain Reaction (RT-PCR)

As was previously described [46], cDNA was used for each 10 µL reaction together with 2× BioRad SSO Advnaced Universal SYBR Green Supermix (BioRad, Hercules, CA, USA) which included buffer, Taq DNA polymerase, dNTPs and SYBR green dye. Specific primers (forward and reverse (Table 2) and cDNA or water (for no template control) were added to each PCR reaction. The specific primers used can be seen in Table 2. For each gene, the optimal MgCl_2_ concentration produced the amplification plot with the lowest cycle product (Cp), the highest fluorescence intensity and the steepest amplification slope. Master mix (8 µL) was pipetted into the 96-well plate and 2 µL cDNA was added as PCR template. Each run contained seven standard curve points in duplicate. A no template control of nuclease-free water was included to exclude DNA contamination in the PCR mix. The double stranded DNA was amplified in the Bio-Rad CFX96 Touch (Bio-Rad Laboratories, Hercules, CA, USA) using the following PCR conditions: initial denaturing at 95 °C for 30 s, 40 cycles of denaturing at 95 °C for 15 s, various annealing temperatures according to Integrated DNA Technologies (IDT) for 30 s and elongating at 60 °C for 30 s. The data on the expression levels of the genes were obtained as Cp values based on the “second derivative maximum” (automated method) as computed by the software. For each of the 12 genes, the reactions were run in duplicate. All assays were quantified by including a standard curve in the real-time qPCR analysis. The next four points of the standard curve were prepared by a 1:10 dilution. Each point of the standard curve was included in duplicate. A graph of Cp vs. log 10 concentrations was produced by the software and the efficiencies were calculated as 10[1/slope]. The specificity of the amplified real-time RT-PCR products were verified by melting curve analysis (60–95 °C) after 40 cycles, which should result in a number of different specific products, each with a specific melting temperature. In addition, we electrophoresed the resulting PCR products on a 2%-agarose gel, stained the gel with ethidium bromide, and visualized it under UV light. PCR-positive products were purified of primer dimers and other non-specific amplification by-products using QIAquick Gel Kit (Qiagen Inc., Valencia, CA, USA) prior to sequencing. We sequenced the products using BigDye^®^ Terminator v3.1 Cycle Sequencing Kits (Applied Biosystems, Foster City, CA, USA) and ABI Automated 3430xl DNA Analyzer (Applied Biosystems) and analyzed them with Sequencing Analysis ver. 5.2 (Applied Biosystems). We aligned sequences of hepcidin with those from related organisms obtained from Gen Bank using a basic alignment-search tool (BLAST; National Center for Biotechnology Information, Bethesda, MD, USA). Sequence alignments were performed for all samples. We used the ClustalW program for sequence alignment. 

### 2.12. 16S rRNA Gene Amplification and Sequencing

Microbial genomic DNA was extracted from cecal samples using the PowerSoil DNA isolation kit, as described by the manufacturer (MoBio Laboratories Ltd., Carlsbad, CA, USA). Bacterial 16S rRNA gene sequences were PCR-amplified from each sample using the 515F-806R primers for the V4 hypervariable region of the 16S rRNA gene, including 12-base barcodes, as previously published [47]. PCR procedure reactions consisted of 25 μL Primestar max PCR mix (Takara Kusatsu, Shiga, Japan), 2 μM of each primer, 17 µL of ultra-pure water, and 4 µL DNA template. Reaction conditions consisted of an initial denaturing step for 3 min at 95 °C followed by 30 cycles of 10 s at 98 °C, 5 s at 55 °C, 20 s at 72 °C, and final elongation at 72 °C for 1 min. PCR products were then purified with Ampure magnetic purification beads (Beckman Coulter, Atlanta, GA, USA) and quantified using a Quant-iT PicoGreen dsDNA quantitation kit (Invitrogen, Carlsbad, CA, USA). Equimolar ratios of total samples were pooled and sequenced at the Faculty of Medicine of the Bar Ilan University (Safed, Israel) using an Illumina MiSeq Sequencer (Illumina, Inc., Madison, WI, USA).

### 2.13. 16S rRNA Gene Sequence Analysis

Data analysis was performed using QIIME2 [48]. Sequence reads were demultiplexed by per-sample barcodes and Illumina-sequenced amplicon reads errors were corrected by Divisive Amplicon Denoising Algorithm (DADA2) [49]. A phylogenetic tree was generated and sequences were classified taxonomically using the Greengenes [50] reference database at a confidence threshold of 99%. The Greengenes taxonomies were used to generate summaries of the taxonomic distributions of features across different levels (phylum, order, family, and genus). Alpha and beta diversity analysis were calculated based on a feature table with samples containing at least 7026 sequences. Richness and evenness, alpha diversity parameters, were calculated using the Faith’s Phylogenetic Diversity and Pielou’s Evenness measures [51]. Beta diversity was analyzed using weighted and unweighted UniFrac distances [52]. Linear discriminant analysis Effect Size (LEfSe) [53] was used to determine the features significantly differ between samples according to relative abundances.

Metagenome functional predictive analysis was carried out using phylogenetic investigation of communities by reconstruction of unobserved states (PICRUSt) [54] software (version 1.1.3). Briefly, feature abundance was normalized by 16S rRNA gene copy number, identified and compared to a phylogenetic reference tree using the Greengenes database, and was assigned functional traits and abundance based on known genomes and prediction using the Kyoto Encyclopedia of Genes and Genomes (KEGG). Data representing significant fold-change differences in functional pathways between experimental groups was plotted.

### 2.14. Morphological Examination

As was previously described [26,46], intestinal samples (duodenal region as the main intestinal Fe absorption site) were collected at the conclusion of the study and from each treatment group. Samples were fixed in fresh 4% (*v*/*v*) buffered formaldehyde, dehydrated, cleared, and embedded in paraffin. Serial sections were cut at 5 µm and placed on glass slides. Sections were deparaffinized in xylene, rehydrated in a graded alcohol series, stained with hematoxylin and eosin, and examined by light microscopy. Morphometric measurements of villus height, width and goblet cell diameter were performed with a light microscope using EPIX XCAP software (Standard version, Olympus, Waltham, MA, USA). 

### 2.15. Statistical Analyses

The in vivo and in vitro results were analyzed by ANOVA using the general linear models procedure of SAS software (version 9.4, SAS Institute Inc., Cary, NC, USA), and differences between treatment groups were compared by using the Student’s *t*-test and values were considered statistically different at *p* < 0.05 (values in the text are means ± SEM). For the microbiome results, the Faith’s Phylogenetic Diversity and Pielou’s Evenness measures difference between groups were analyzed by Kruskal–Wallis (pairwise) test. Differences between Weighted/Unweighted UniFrac distances were analyzed by Pairwise permanova test. Analysis of composition of microbiomes (ANCOM) is a bioinformatics method to identify features that are differentially abundant (i.e., present in difference abundances) across sample groups. Significant *p*-values (*p* < 0.05) associated with microbial clades and functions identified by LEfSe were corrected for multiple comparisons using the Benjamini Hochberg false discovery rate (FDR) correction. Statistical analysis was performed using SAS version 9.3 (SAS Institute, Cary, NC, USA). The level of significance was established at *p* < 0.05.

## 3. Results

### 3.1. Phytate Concentration and Polyphenol Profile in the Bean Flours

The concentration of the five most prevalent polyphenolic compounds found in the bean seed coats is presented in Table 3. The Fe-standard beans (BRS Perola) presented higher (*p* < 0.05) concentration of epicatechin and quercetin 3-glucoside compared to the Fe-biofortified beans (BRS Cometa). There was no difference (*p* > 0.05) in the phytate (*n* = 5) concentration between the Fe-biofortified and Fe-standard carioca bean flour.

### 3.2. Dietary Fiber and Protein Concentration in the Bean Flours

There was no difference (*p* > 0.05) in the insoluble, soluble and total dietary fiber. However, the protein concentration is higher (*p* < 0.05) in the Fe-biofortified bean (BRS Cometa) compared to the Fe-standard bean (BRS Perola) (Table 4). 

### 3.3. In Vitro Assay (Caco-2 Cell Ferritin Formation)

Ferritin, the cellular Fe storage protein was used as an indicator of Fe bioavailability [42,43]. Ferritin concentrations were significantly higher in cells exposed to the Fe-biofortified (BC) bean based diet versus the Fe-standard (SC) bean based diet (*p* < 0.05, *n* = 6, Table 5). These results indicate greater amounts of bioavailable Fe in the Fe-biofortified bean based diet. 

### 3.4. In Vivo Assay (Gallus Gallus Model)

#### 3.4.1. Growth Rates, Hb, Hb-Fe, and HME

**Figure 1:**
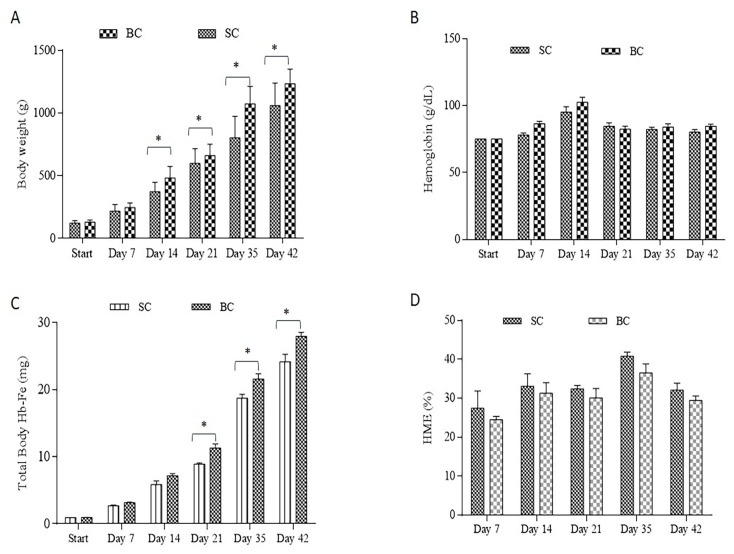
Fe-related parameters assessed during the study. (**A**): Body weight (g); (**B**) Blood hemoglobin concentration (g/L); (**C**): Total body Hb-Fe (mg); (**D**): Hemoglobin maintenance efficiency (%). Values are means ± SEM. * Statistical difference by *t*-test at 5% of probability. SC: Fe-standard carioca bean diet; BC: Fe-biofortified carioca bean diet.

The feed intake and the Fe intake were higher (*p* < 0.0001) in the BC group (average consumption of 56.49 g diet/day ± 0.8) and cumulative (day 42) Fe intake of 111.3 mg Fe ± 1.5, compared to the SC group (average consumption of 51.06 g diet/day ± 2.1), and cumulative (day 42) Fe intake of 86.9 mg Fe ± 3.3. In addition, as from day 14 of the study, body weights were consistently higher (*p* < 0.05) in the group receiving the Fe-biofortified bean diet versus the group receiving the standard bean diet (Figure 1A). There were no significant differences (*p* > 0.05) in the hemoglobin concentrations between the treatments at any time point (Figure 1B). As from day 21, the total body Hb-Fe was significantly greater in the group receiving the Fe-biofortified carioca bean (*p* < 0.05, Figure 1C). However, no differences in HME values were measured between the groups (*p* > 0.05, Figure 1D)

#### 3.4.2. Gene Expression of Fe—Related and BBM Functional Proteins 

**Figure 2:**
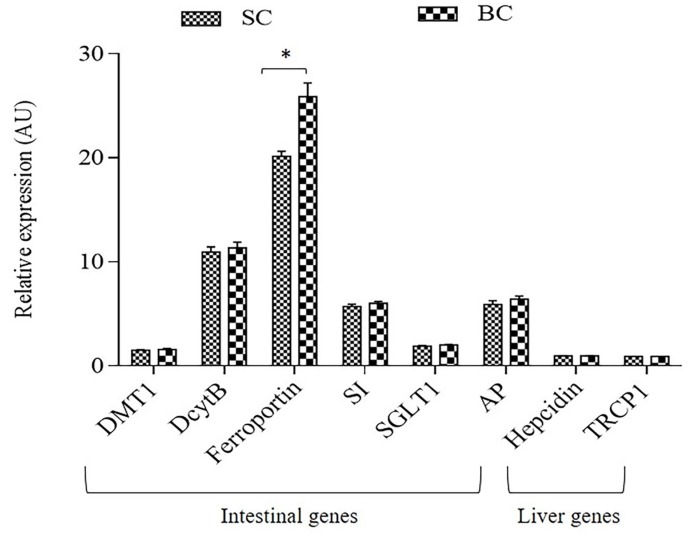
Duodenal and liver mRNAgene expression of Fe-related proteins collected on day 42. Changes in mRNA expression are shown relative to expression of 18S rRNA in arbitrary units (AU, * *p* < 0.05). SC: Fe-standard carioca bean diet; BC: Fe-biofortified carioca bean diet; DMT1: Divalent Metal Transporter 1; DcytB: Duodenal cytochrome b; SI: Sucrose isomaltase; SGLT1: Sucrose isomaltase 1; AP: Amino peptidase; TRCP1: Transferrin Receptor Protein 1.

Relative to 18S rRNA, duodenal gene expression of ferroportin was significantly elevated (*p* < 0.05) in the group receiving the Fe-biofortified carioca bean based diet (BC) (Figure 2). However, no significant differences (*p* > 0.05) in the expression of the other Fe-related proteins were observed between treatment groups (Figure 2).

#### 3.4.3. Morphometric Measurements

**Figure 3:**
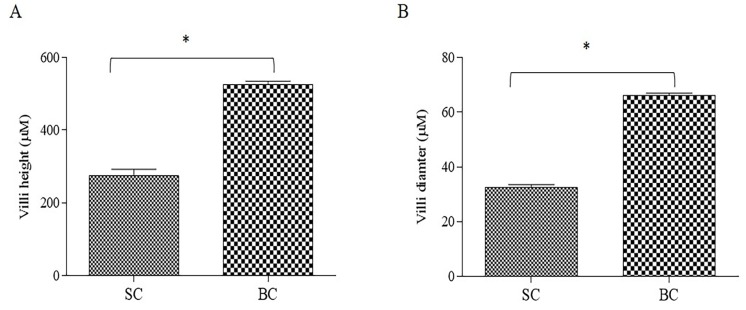
Effect of Standard and Biofortified diets on the duodenal small intestinal parameters: (**A**) Intestinal villi height (μM); (**B**) Intestinal villi diameter. SC: Fe-standard carioca bean diet; BC: Fe-biofortified carioca bean diet. Values are means ± SEM, *n* = 5. * Statistical difference by *t*-test (*p* < 0.0001).

The BC group presented higher (*p* < 0.0001) villi height (Figure 3A) and diameter (Figure 3B) compared to the SC group. This serves as a mechanical measurement of brush border membrane absorptive ability and improvement in brush border membrane functionality and overall gut health [46]. It indicates that the consumption of Fe biofortified carioca beans could lead to a proliferation of enterocytes.

There were no significant differences (*p* > 0.05) in goblet cells (mucus producing and secreting cells) number per intestinal villi. However, the goblet cell diameter was slightly higher (*p* < 0.05) in the SC group (4.74 μM ± 2.03) compared to the BC group (4.56 μM ± 1.84).

#### 3.4.4. Microbial Analysis

Comparisons were made between Fe-biofortified carioca bean diet (BC) and Fe-standard carioca bean diet (SC) beans groups. Cecal contents samples from the standard and biofortified varieties were collected and used for bacterial DNA extraction and sequencing of the V4 hypervariable region in the 16S rRNA gene. The contents of the cecum highly diverse and abundant microbiota and represent the primary site of bacterial fermentation [55]. 

**Figure 4:**
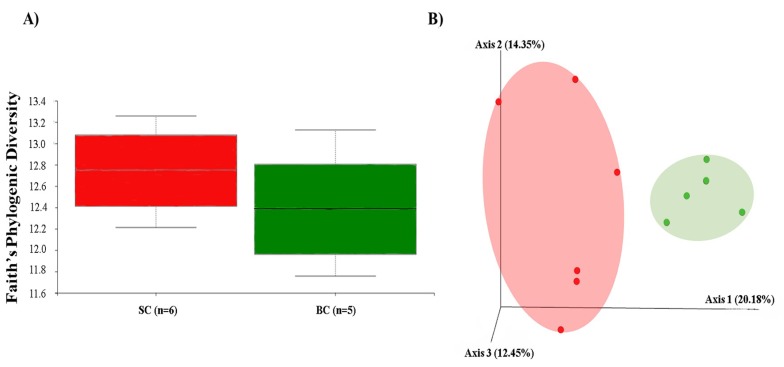
Microbial diversity of the cecal microbiome in Carioca diet. SC: Fe-standard carioca bean diet; BC: Fe-biofortified carioca bean diet. (**A**) Measure of α-diversity using the Faith’s Phylogenetic Diversity; and (**B**) Measure of β-diversity using unweighted UniFrac distances separated by the first three principal components (PCoA). Each dot represents one animal, and the colors represent the different treatment groups within Carioca beans (red = SC; green = BC).

The diversity of the cecal microbiota between the standard carioca bean (SC) and biofortified carioca bean (BC) was assessed initially through measures of α and β-diversity. Faith’s phylogenetic diversity, used to assess α-diversity (Figure 4A), was not significant between SC and BC groups (*p* > 0.05). We utilized unweighted UniFrac distances as a measure of β-diversity to assess the effect of BC diet on between-individual variation in bacterial community (Figure 4B). Principal coordinate analysis showed statistically significant difference in clustering between the BC and SC groups, suggesting that individual samples were more similar to other samples within the same group, as opposed to samples of the other group (*p* > 0.05). Furthermore, individual samples of the BC group clustered significantly closer to each other than did members of the SC group (*p* < 0.05). 

**Figure 5:**
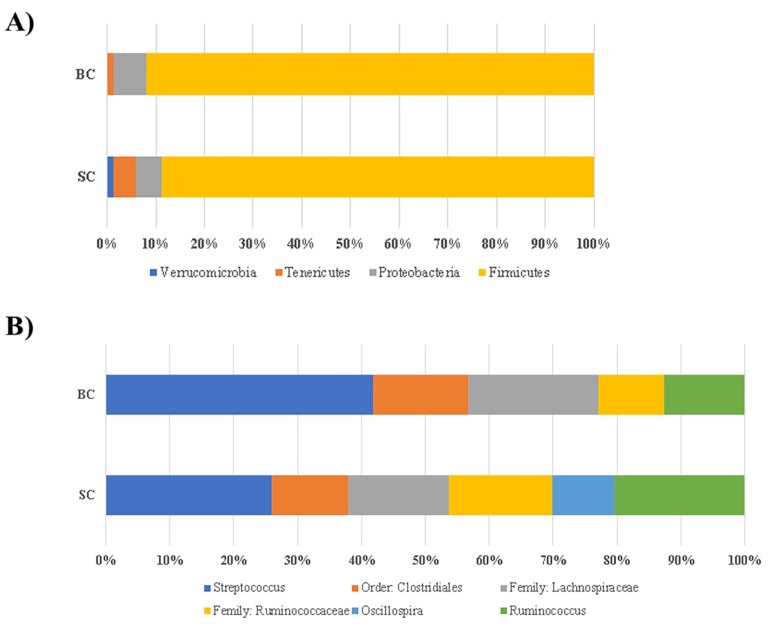
Compositional changes of gut microbiota in response to a Carioca standard versus biofortified diet. SC: Fe-standard carioca bean diet; BC: Fe-biofortified carioca bean diet. (**A**) Phylum level changes in the BC and SC groups as measured at the end of the study (day 42). Only phyla with abundance ≥1% are displayed; (**B**) Genus level changes in the BC and SC groups as measured at the end of the study (day 42). Only genera with abundance ≥5% are displayed.

Following α and β-diversity, we conducted a taxon-based analysis of the cecal microbiota. 16S rRNA gene sequence revealed that >98% of all bacterial sequences in both treatment groups of the carioca variety. Both of the treatment groups were dominated by two major phyla: Firmicutes and Proteobacteria, whereas sequences of Tenericutes and Verrucomicrobia were also identified, but in much lower abundance. After FDR correction, there were no significant differences between groups at the genus level for the carioca variety (Figure 5A,B). As in the human gut [56], the Firmicutes phyla vastly predominated in the *Gallus gallus* cecum [57]. 

**Figure 6:**
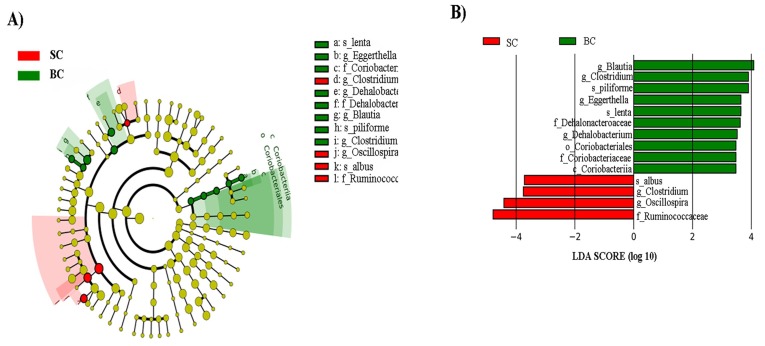
LEfSe method identifying the most differentially enriched taxa in the Standard and Biofortified Carioca diet groups. SC: Fe-standard carioca bean diet; BC: Fe-biofortified carioca bean diet. (**A**) Taxonomic cladogram obtained using LEfSe analysis of the 16S rRNA sequences. Treatment groups are indicated by the different colors, with the brightness of each dot proportional to its effect size; (**B**) Computed LDA (Linear discriminant analysis) scores of the relative abundance difference between the standard Carioca bean diet and the biofortified Carioca bean diet. Negative LDA scores (red) are enriched in standard Carioca bean diet while positive LDA scores (green) are enriched in biofortified Carioca beans.

The final analysis investigation of relative abundances at all taxonomic levels with carioca beans was carried out using the linear discriminant analysis effect size (LEfSe) method to investigate significant bacterial biomarkers that could identify differences in the gut microbiota of SC and BC groups [53]. Figure 6A,B present the differences in abundance between groups at the various taxonomic levels, with their respective LDA (Linear discriminant analysis) scores. We observed a general taxonomic delineation between the SC and BC groups, whereby the SCFA-producing Firmicutes predominated in the BC groups. Specifically, *Eggerthella lenta* (LDA score = 3.65, *p* = 0.011) and *Clostridium piliforme* (LDA = 3.90, *p* = 0.006); there were members of the *Coriobacteriaceae* (LDA = 3.65, *p* = 0.011), *Dehalobacteriaceae* (LDA = 3.52, *p* = 0.044), *Lachnospiraceae* (LDA = 3.90, *p* = 0.006) were significantly enriched in the BC group. In the SC group, however, members of the Firmicutes, Tenericutes and Proteobacteria were the predominantly-enriched phyla. Specifically, *Ruminococcus albus* (LDA score = 3.72, *p* = 0.017), and members of the *Oscillospira* (LDA score = 4.41, *p* = 0.044) and *Clostridium* (LDA score = 3.75, *p* = 0.006) genera were significantly enriched in the SC group. 

## 4. Discussion

In studies of Fe biofortification, there is a clear need and advantage to have in place screening tools capable of evaluating biofortified lines of staple food crops, both individually and in the context of the diet for which they are consumed [4,7,36]. The present study, therefore evolved as an opportunity to demonstrate how the in vitro digestion/Caco-2 cell model and the *Gallus gallus* in vivo model of Fe bioavailability could be applied in the design of an Fe bioavailability study aimed at assessing the Fe bioavailability of Fe biofortified versus standard carioca beans. The diets that were used were specifically formulated according to the Brazilian dietary survey [45] (Table 1). Overall, the data presented in this manuscript are in agreement with previously published research [4,19,22], indicating that this dual in vitro/in vivo screening approach is effective in the assessment of Fe bioavailability of Fe biofortified beans. 

The in vivo results showed that although Hb levels were not significantly increased in the Fe biofortified carioca bean group, significant differences in total body Hb-Fe, a sensitive biomarker of dietary Fe bioavailability and status [58], were observed starting on week four of the study (Figure 1), indicating on an improvement in Fe status in the Fe biofortified group. In addition, the animals receiving the standard bean variety had a higher HME at each time point when compared to the group receiving the Fe biofortified carioca beans, indicating an adaptive response (e.g., a relative up-regulation of absorption) to less absorbable dietary Fe [4,22,23,39]. The Fe-biofortified carioca bean diet presented higher Fe content and lower PA: Fe ratio compared to the Fe-standard carioca bean diet (Table 1), which could contribute to the higher dietary Fe bioavailability of this group [59,60,61]. 

Additionally, the in vitro assay (Table 5) further supported the in vivo findings. Ferritin values in cells exposed to the Fe-biofortified bean variety only, were low and similar to ferritin values in cells exposed to the standard bean variety only. In contrast, once the Fe biofortified bean variety was included in the experimental bean based diet, an increase in ferritin formation was observed relative to cells exposed to the standard bean based diet. This could be due to the higher Fe content and the lower PA: Fe ratio presented in the Fe-biofortified bean based diet, but can also be attributed to the other dietary ingredients, and their potential effect on dietary Fe bioavailability. 

These results are in agreement with previous studies aimed at assessing the Fe promoting effects of Fe-biofortified black beans [19], red mottled beans [37] and pearl millet [22]. Thus, since a number of intrinsic factors, including polyphenol compounds and phytates, may influence the bioavailability of Fe from these beans and other crops [4,17,20,24], and limit their nutritional benefit. This suggests that increased bean Fe concentration alone may not be sufficient to yield significant physiological improvements in Fe status. In this context, it is important to note that in addition to increased Fe content, the Fe-biofortified bean variety had a higher protein content (*p* < 0.05, Table 4), this may further affect the nutritional benefit of this bean variety. Current results are in agreement with recent research indicating that dietary ingredients as potato may enhance the Fe absorption when consumed with beans, whereas other foods consumed with beans, as rice, might negatively affect Fe bioavailability (in vitro) [11].

Previous studies have shown a higher concentration of polyphenolic compounds (PP) and phytate in the Fe-biofortified beans compared to the Fe-standard beans [11,19,62]. However, in the current study the Fe-biofortified carioca bean presented lower concentrations of some PPs and no difference in the phytate concentration compared to the Fe-standard carioca bean (Table 3). This is an interesting finding since the PPs and phytate are known as strong inhibitors of Fe bioavailability [19,23,24,63]. Thus, this Fe-biofortified variety could be a more effective vehicle for the Fe biofortification program. This point was demonstrated, as the totality of the results indicated that the Fe biofortified carioca bean based diet was moderately effective at increasing the bioavailable and therefore absorbable dietary Fe both in vitro and in vivo.

Further, the duodenal gene expression of ferroportin (FPN) was significantly elevated in the group receiving the Fe-biofortified bean diet (*p* < 0.05, Figure 2). However, no significant differences in the expression of the other Fe-related and brush border membrane functional proteins were observed between treatment groups. In contrast, some studies have shown a down-regulation of the gene expression of these proteins (DMT-1, ferroportin and Dcytb) in Fe-biofortified diets compared to the Fe-standard diets [4,22,37,64]. Ferroportin is an Fe exporter protein that transfer the Fe across the basolateral membrane of the enterocyte [34]. Thus, since the BC group presented a higher expression of FPN, more Fe can be released from the enterocyte into the blood circulation, therefore, this mechanism suggests increased amounts of absorbable Fe, hence, the total body Hb-Fe increased in the Fe biofortified group compared to the standard. 

As is the case in humans and the vast majority of animals, the *Gallus gallus* model harbor a complex and dynamic gut microbiota [65], heavily influenced by host genetics, environment and diet [66]. There is considerable similarity at the phylum level between the gut microbiota of *Gallus gallus* and humans, with Bacteroidetes, Firmicutes, Proteobacteria, and Actinobacteria representing the four dominant bacterial phyla in both [67]. In the current study, a general taxonomic delineation between the SC and BC group was observed, whereby the SCFA-producing Firmicutes predominated in the BC group. Specifically, *Eggerthella lenta* and *Clostridium piliforme* (Figure 6B). The increase in the SCFA-producing bacteria could lead to an increased SCFA concentration in the intestinal lumen, which in return can promote intestinal cell proliferation [68], as was observed in the BC group that presented an increase in duodenal villi height (Figure 3). This observation is in agreement with previous research indicating that duodenal villi height was significantly increased due to dietary fiber (as xylooligosaccharides) that have led to increased SCFA bacterial production in vivo [69]. Also, the Fe-biofortified bean presented a higher, although not significant, soluble fiber content compared to the Fe-standard bean (Table 4). Soluble fiber can increase the villi height by increasing the intestinal cell proliferation [70] (Figure 3). 

In addition, and as was mentioned above, the Fe-biofortified bean presented higher (*p* < 0.05) protein content compared to the Fe-standard bean (Table 4), a higher protein contented in a diet was shown increase villi height and intestinal cell proliferation [71]. Undigested dietary proteins and fibers are fermented in the intestine and this fermentation process produces SCFAs (mainly composed by acetate, propionate, and butyrate). Functionally, SCFAs affect the metabolism and gut health [72]. Acetate and propionate are energy substrates for peripheral tissues and butyrate is preferentially used as an energy source by colonic epithelial cells [73,74]. 

In this study, the abundance of members of the *Coriobacteriaceae*, specially *Eggerthella lenta* and *Lachnospiraceae* were enriched in the BC group (Figure 5B). These results demonstrate a potential beneficial effect of the Fe-biofortified bean diet on the intestinal microbial composition, since these microorganisms can improve the host health [75,76]. *Lachnospiraceae* is a butyrate producer family [75]. This short chain fatty acid (SCFA) is an energy source of colonocytes and it stimulates the immunogenicity of cancer cells [76]. *Coriobacteriacea* acts on the conversion of bile salts and steroid hormones, and the *Eggerthella lenta* was recently found to reductively cleave the heterocyclic C-ring of the epicatechin and catechin [77], and the breakdown product (3-(3,4-dihydroxyphenyl) propionic acid) presents anti-inflammatory effects [78]. This result is especially important since in general, carioca beans present these flavonoids (Table 3), thus they can be metabolized by the bacteria from the BC group. 

Further, one of the aims of this study was to determine whether ingestion of an Fe biofortified diet would lead to an increased pathogenic bacterial load in the gut microbiota. Dietary Fe supplementation has been associated with an inflammatory-promoting gut microbiota, most likely due to the increased presence of luminal Fe [79], subsequent generation of free radicals, and ensuing epithelial stress and microbial dysbiosis [80]. Many of the nutritional methods used to combat Fe deficiency, such as Fe supplementation and Fe fortification, induce dysbiotic conditions and an expansion of pathogenic bacteria in the gut microbiota of subjects receiving Fe replete diets [79,81]. In contrast to these findings, we did not observe significant increase in pathogenic taxa in the BC group that have been previously associated with dietary Fe intake (e.g., Salmonella and other Enterobacteria) [81]. Therefore, this finding suggests that the use of biofortified beans instead of Fe fortification or Fe supplementation can be an effective and potentially sustainable strategy to reduce the Fe deficiency, with additional improvement in the gut bacterial populations. 

Overall, we demonstrate in vitro that the potential consumption of the Fe-biofortified bean in a food basket context may increase the Fe uptake. The in vivo analyses demonstrated a significant remodeling of the gut microbiota in animals receiving a Fe-biofortified diet, which also presented higher amount protein. This microbiota remodeling increased the SCFA-producing bacteria abundance, improving the morphometric parameters (villi height), and increasing the intestinal absorptive surface area, these findings can potentially lead to increased Fe bioavailability and uptake. Therefore, under these experimentalal conditions, the results suggest that the consumption of the Fe biofortified carioca bean with other staple foods (i.e., food basket), increased Fe bioavailability, improved Fe status, and improved the composition and function of the gut microbiota. Understanding the effect of Fe biofortification on the gut microbiota may help to further biofortification efforts by improving the safety and efficacy profile of the food crop, as we understand more about the relationship between biofortified diets and the resident gut microbiota. 

## 5. Conclusions

Nutritional methods aimed to alleviate global Fe deficiency, such as Fe supplementation or Fe fortification, have been moderately efficacious at attaining optimal Fe status. However, any improvement in serum Fe levels comes at the expense of decreased gut health in the form of dysbiosis and infection. This study showed how Fe-biofortification affects the composition and metagenome of the gut microbiota and intestinal function. Animals (*Gallus gallus*) that consumed the Fe biofortified carioca bean-based diet had less abundance of pathogenic bacteria, with concomitant increases in SCFA-producing bacteria that have known phenolic catabolic capacity, which have led to an improvement in intestinal morphology. In addition, and for the first time, the Fe-biofortified carioca bean presented similar concentration of phytate and polyphenols, yet, a higher protein content, in comparison to the Fe-standard bean, which potentially can increase the Fe bioavailability, and intestinal functionality, respectively. 

Further and similar to previous data, the current research suggests that increased Fe content may not necessarily result in an increased absorbable Fe, and a key factor is the measurement of dietary Fe bioavailability in Fe biofortified crop varieties based diets, and as part of the breeding process.

Collectively, the findings presented here provide evidence that, unlike other nutritional methods of increasing Fe status, the Fe biofortification appear to improve the gut microbiota, and they raise the possibility that this strategy can further improve the efficacy and safety of the crop Fe biofortification approach. We suggest the utilization of the discussed in vitro and in vivo screening tools to guide studies aimed to develop and evaluate Fe biofortified staple food crops, and their potential nutritional benefit. 

## Figures and Tables

**Table 1:** Composition of the experimental bean based diets ^1–3^.

Ingredient	Fe Content (μg Fe/g Sample)	Fe-Standard Carioca Based Diet (SC) (g/kg by Formulation)	Fe-Biofortified Carioca Bean Based Diet (BC) (g/kg by Formulation)
BRS Perola (Fe-standard bean)	64.3 ± 0.54	420	-
BRS Cometa (Fe-biofortified bean)	84.97 ± 2	-	420
Potato	12.89 ± 0.43	320	320
Corn	31.36 ± 4.74	70	70
Pasta (non-enriched)	13.82 ± 1.04	70	70
Rice	4.21 ± 0.8	50	50
Vitamin/mineral premix (no Fe)	0.0	70	70
dl-Methionine	0.0	2.5	2.5
Vegetable oil	0.0	30	30
Choline chloride Total (g)	0.0	0.75	0.75
Total (g)			
**Selected components**			
Dietary Fe concentration (μg/g)	-	40.47 ± 1.84	47.04 ± 1.52 *
Phytic acid (μg/g)	-	1.71 ± 0.16 *	1.15 ± 0.053
Phytate:Fe molar ratio	-	35.76	20.84

^1^ Vitamin and mineral premix provided/kg diet (330002 Chick vitamin mixture; 235001 Salt mix for chick diet; Dyets Inc. Bethlehem, PA, USA). ^2^ Iron concentrations in the diets were determined by an inductively-coupled argon-plasma/atomic emission spectrophotometer. ^3^ Method for determining phytate is described in the materials and method s section. * Statistical difference by *t*-test at 5% of probability (Comparison between Standard diet and Biofortified diet).

**Table 2:** DNA sequences of the primers used in this study.

Analyte	Forward Primer (5′-3′) (Nucleotide Position)	Reverse Primer (5′-3′)	Base Pairs Length	GI Identifier
*Iron metabolism*				
DMT1	TTGATTCAGAGCCTCCCATTAG	GCGAGGAGTAGGCTTGTATTT	101	206597489
Ferroportin	CTCAGCAATCACTGGCATCA	ACTGGGCAACTCCAGAAATAAG	98	61098365
DcytB	CATGTGCATTCTCTTCCAAAGTC	CTCCTTGGTGACCGCATTAT	103	20380692
Hepcidin	AGACGACAATGCAGACTAACC	CTGCAGCAATCCCACATTTC	132	
TRCP1	GAGCAAGCCATGTCAAGATTTC	GTCTGGGCCAAGTCTGTTATAG	122	015291382.1
*BBM functionality*				
SI	CCAGCAATGCCAGCATATTG	CGGTTTCTCCTTACCACTTCTT	95	2246388
SGLT1	GCATCCTTACTCTGTGGTACTG	TATCCGCACATCACACATCC	106	8346783
AP	CGTCAGCCAGTTTGACTATGTA	CTCTCAAAGAAGCTGAGGATGG	138	45382360
18S rRNA	GCAAGACGAACTAAAGCGAAAG	TCGGAACTACGACGGTATCT	100	7262899

DMT-1, Divalent Metal Transporter–1; DcytB, Duodenal cytochrome b; 18S rRNA, 18S Ribosomal subunit; SI, Sucrose isomaltase; SGLT-1: Sodium-Glucose transport protein 1; AP, Amino peptidase; TRCP1: Transferrin Receptor Protein 1; BBM, Brush border membrane.

**Table 3:** Phytate concentration and polyphenol profile (μM) present in common bean flours.

Food Flours	Phytate (g/100 g)	Polyphenol Profile				
		Kaempferol 3-Glucoside	Catechin	Epicatechin	Procyanidin B1	Quercetin 3-Glucoside
BRS Perola (Fe-standard)	1.05 ± 0.03	17.3 ± 1	26.1 ± 1.3	12.8 ± 1.7 *	1.4 ± 0.2	0.2 ± 0.1
BRS Cometa (Fe-biofortified)	1.08 ± 0.005	16.2 ± 1.1	25.9 ± 4.6	11 ± 1.4	1.2 ± 0.2	-

Values are means ± SEM. * Statistical difference by *t*-test at 5% of probability (*p* = 0.0451).

**Table 4:** Dietary fiber and protein concentration in the beans (g/100 g).

Beans	Insoluble Fiber	Soluble Fiber	Total Fiber	Total Protein
BRS Perola (Fe-standard)	20.80 ± 0.02	3.77 ± 1.03	24.56 ± 1.05	24.15 ± 0.44
BRS Cometa (Fe-biofortified)	18.71 ± 0.94	4.85 ± 0.33	23.55 ± 1.27	29.01 * ± 0.29

* Statistical difference by *t*-test (*p* = 0.0001).

**Table 5:** Ferritin concentration in Caco-2 cells exposed to samples of bean based diets, and additional meal plan ingredients ^1−2^.

Tested Sample	Ferritin (ng/mg of Protein)
*Tested Diets*	
Standard Fe diet (SC) (40.4 ± 1.8 µg/g diet)	5.04 ± 0.37
Biofortified Fe diet (BC) (47.0 ± 1.5 µg/g diet)	6.10 ± 0.29 *
*Ingredients*	
BRS Perola (Fe-standard bean) (64.3 ± 0.5 µg/g bean)	7.87 ± 1.15 ^d^
BRS Cometa (Fe-biofortified bean) (84.9 ± 2 µg/g bean)	5.74 ± 0.34 ^d^
Potato flour (12.8 ± 0.4 µg/g flour)	21.74 ± 0.83 ^a^
Pasta flour (13.8 ± 1.0 µg/g flour)	12.79 ± 0.60 ^b^
Corn flour (31.3 ± 4.7 µg/g flour)	10.38 ± 0.94 ^c^
Rice flour (4.2 ± 0.8 µg/g flour)	6.12 ± 1.02 ^d^

^1^ Caco-2 bioassay procedures and preparation of the digested samples are described in the materials and methods sections. ^2^ Cells were exposed to only MEM (minimal essential media) without added food digests and Fe (*n* = 6). All samples were run in the same experiment. ^a–d^ Values are means ± SEM. Different letters indicate statistical differences at 5% by Newman–Keuls test. * Indicates statistical differences at 5% by t-test between the experimental diets.
